# A Review on Pathophysiology, and Molecular Mechanisms of Bacterial Chondronecrosis and Osteomyelitis in Commercial Broilers

**DOI:** 10.3390/biom13071032

**Published:** 2023-06-23

**Authors:** Venkata Sesha Reddy Choppa, Woo Kyun Kim

**Affiliations:** Department of Poultry Science, University of Georgia, Athens, GA 30602, USA

**Keywords:** broiler, lameness, bacterial chondronecrosis and osteomyelitis (BCO), biomarkers, mesenchymal cells

## Abstract

Modern day broilers have a great genetic potential to gain heavy bodyweights with a huge metabolic demand prior to their fully mature ages. Moreover, this made the broilers prone to opportunistic pathogens which may enter the locomotory organs under stress causing bacterial chondronecrosis and osteomyelitis (BCO). Such pathogenic colonization is further accelerated by microfractures and clefts that are formed in the bones due to rapid growth rate of the broilers along with ischemia of blood vessels. Furthermore, there are several pathways which alter bone homeostasis like acute phase response, and intrinsic and extrinsic cell death pathways. In contrast, all the affected birds may not exhibit clinical lameness even with the presence of lameness associated factors causing infection. Although *Staphylococcus*, *E. coli*, and *Enterococcus* are considered as common bacterial pathogens involved in BCO, but there exist several other non-culturable bacteria. Any deviation from maintaining a homeostatic environment in the gut might lead to bacterial translocation through blood followed by proliferation of pathogenic bacteria in respective organs including bones. It is important to alleviate dysbiosis of the blood which is analogous to dysbiosis in the gut. This can be achieved by supplementing pro, pre, and synbiotics which helps in providing a eubiotic environment abating the bacterial translocation that was studied to the incidence of BCO. This review focused on potential and novel biomarkers, pathophysiological mechanism, the economic significance of BCO, immune mechanisms, and miscellaneous factors causing BCO. In addition, the role of gut microbiomes along with their diversity and cell culture models from compact bones of chicken in better understanding of BCO were explored.

## 1. Introduction

An active tissue that experiences continual remodeling is the bone. Any changes in its regular turnover may lead to skeletal diseases characterized by bone loss [1]. During the past few decades, commercial poultry production increased dramatically in terms of feed efficiency and growth rate; however, this pattern also showed some adverse implications, such as fatty liver syndrome, pulmonary hypertension, and skeletal problems [2,3,4,5]. Furthermore, modern broilers have the genetic potential to achieve higher body weights with high metabolic demands. This makes them prone to skeletal damage followed by opportunistic bacterial infection and later with bacterial chondronecrosis and osteomyelitis (BCO). The physiology of the bone and its turnover process is complex and involves several pathways working coherently [6]. A common skeletal disease condition that affects broilers worldwide is lameness and is associated with the factors including genetic traits, infectious agents, the center of gravity of the bird, body conformation, activity, and nutrition [7,8,9]. Furthermore, the rate of culling at farm level due to lameness is 0.5% to 4%, which reflects the losses of approximately $100 million per year in United States alone [9,10]. Moreover, BCO is considered as a common cause of lameness in Australia, Canada, Europe, and the US [7,11,12,13,14]. Additionally, BCO in broiler production would impact the poultry revenue through the culling and condemnation rates along with a lowered livability since the production is usually expressed as production costs, net profit per pound of a bird [15,16]. The above impact would be considerably contributed to by the incidence of lame birds in the farm, which causes higher feed conversion, lower weight gain, and higher condemnation rates in processing plant [17]. The current review pivots on BCO, which is the most common cause of leg disorders which raised concerns on animal welfare and economic losses.

## 2. Inflammation and BCO

**Figure 1:**
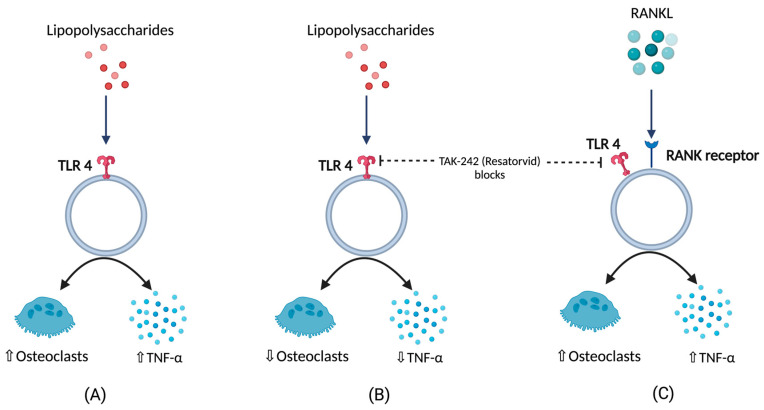
(**A**) Response of lipopolysaccharides (LPS) with respect to TNFα (tumor necrosis factor), and osteoclastic activity (**B**) TNFα, and osteoclastic activity in the presence LPS under the influence of TLR4 blockers (**C**) TNFα, and osteoclastic activity in the presence of RANKL under the influence of TLR4 blockers.

BCO is commonly observed in femurs, tibia, and thoracic vertebrae [10,18]. This occurs majorly due to formation of microfractures and clefts because of rapid growth of juvenile bones. Additionally, this is often associated with rapid increases in body weight, leading to focal ischemia providing a convenient breeding ground for bacterial colonization [18,19]. Post infection or injury, acute phase response (APR) is a key sequela that affects the nutrient requirements and metabolism, and it is usually initiated by local inflammatory response [20]. The above response can be measured by observing the changes in acute phase proteins and cytokine profiles where the excessive levels indicates decreasing production traits and rise in pathology [20,21,22,23,24]. Cytokines (IL-1,6 and tumor necrosis factor) released as a result of APR hasten bone resorption [25,26,27] as well as muscle breakdown [28]. A study involving the injection of lipopolysaccharide (LPS) to the birds showed a severe disruption in bone homeostasis, production parameters including livability, bodyweight, and feed conversion [27]. This usually occurs due to macrophages and osteoclast like cells responding to LPS by release of cytokines and nitric oxide [29]. Moreover, a study on RAW 264.7 cells in vitro were not able to differentiate into mature osteoclasts in the presence of LPS. RANKL or LPS-treated cells increased Toll-like receptor 4 (TLR4) levels in membrane [30]. A TLR4 inhibitor, TAK-242 (resatorvid) reduced the osteoclast number as well as tumor necrosis factor (TNF)-α in LPS treated cells. In contrast, RANKL- induced cells were not affected by TAK-242 and secreted basal levels of TNF-α. This clearly shows that LPS associated bone resorption is associated with LPS/TLR4/tumor necrosis factor receptor (TNFR)-2 axis but not with RANKL/RANK/OPG axis [30]. Furthermore, osteoclasts and their activities are regulated by osteoblasts which, in turn, are altered by the bacteria and their products by means of apoptosis which occurs by activation of intrinsic and extrinsic cell death pathways leading to disruption in bone homeostasis [31] (Figure 1).

## 3. *Pathogens* and BCO

*Staphylococcus aureus* is the most commonly reported organism found in BCO epiphyseal lesion, but septicemic *pathogens* like *Staphylococcus hyicus*, *S. xylosus*, *S. simulans*, *Mycocaterium avium*, *Salmonella* spp., *E. coli*, and *Enterococcus* were also isolated [10,13,32,33]. Histological changes in epiphyseal region of growth plate due to BCO lead to transection of the capillaries and blood vessels within the highly vascularized epiphyseal region, which paves the way for decreased blood flow to the areas around that region [18]. Furthermore, this allows the circulating bacteria to enter and proliferate, along with immunological response leading to tissue damage (necrotic abscesses and voids) [14,18,19,34]. Physiological stress also aids in heightened entry of opportunistic *pathogens* through the epithelial tight junctions, and the pathogens finally arrive at osteochondrotic micro-fractures and clefts [35]. BCO is also observed in clinically non-lame birds at higher incidences, but there are notable pathognomonic lesions seen in lame birds with BCO [14,34]. 

**Figure 2:**
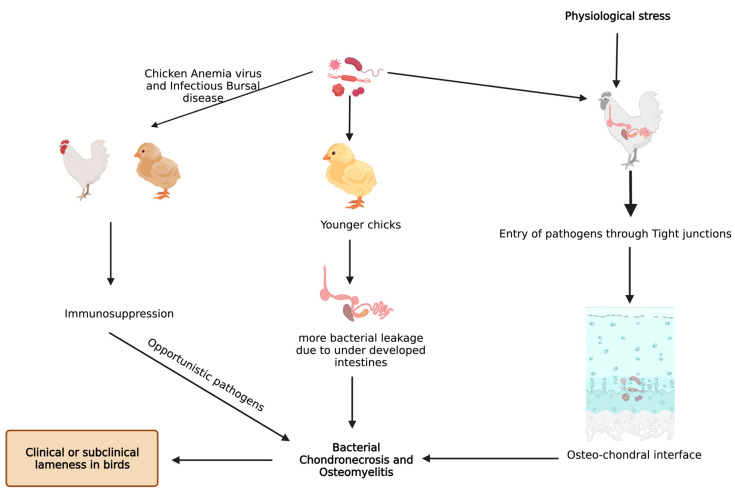
Known pathways through which bacterial chondronecrosis and osteomyelitis (BCO) is observed in modern day broilers.

Bacterial translocation through the damaged epithelium is reported to be one of the causes for higher incidence of BCO [9]. In addition, young intestines have higher susceptibility to bacterial leakage than fully developed intestines in the presence of mucosal damage [9,34]. Furthermore, chicken anemia virus and infectious bursal disease virus make the birds more prone to BCO as a result of immunosuppression [10,36]. An understanding of bacterial diversity, 3D structural alterations in bone and cartilage, bone remodeling marker gene expression, and omega 3 fatty acid and/or probiotic supplementation in BCO is still an important topic that is focused on; however, it is necessary to be further rationalized to identify precise etiology and treatment. Additionally, there is a limited information on extra intestinal bacteria which may induce the APR followed by BCO. In a study involving linear discriminant analysis effect size (LEfSe), an analysis showed that physiological stress will allow commensal and pathogenic bacteria to enter extra intestinal sites, indicating the need for exploring the novel taxa [37,38]. These extra intestinal sites would be circulating maternal blood microbiota in chick or in vivo colonies of microbes [37]. Apart from microbiota, some managemental practices like light intensity, drinking water, and flooring may also affect the incidence of BCO [39]. Light intensity is directly related to the bird movement and activity which may affect the incidence of BCO [40,41,42,43], and a study showed that detecting surface temperatures of broiler leg regions with the help of non-invasive methods would also help in detecting lesions of BCO [44]. In two different studies, provision of 25-OH vitamin D3, prophylactic administration of probiotics were reported to abate the incidence of BCO in wire flooring model which were attributed to trigger the lameness in broilers [34,45] (Figure 2).

## 4. Underlying Anatomy and Physiology behind BCO and Its Potential Biomarkers

**Figure 3:**
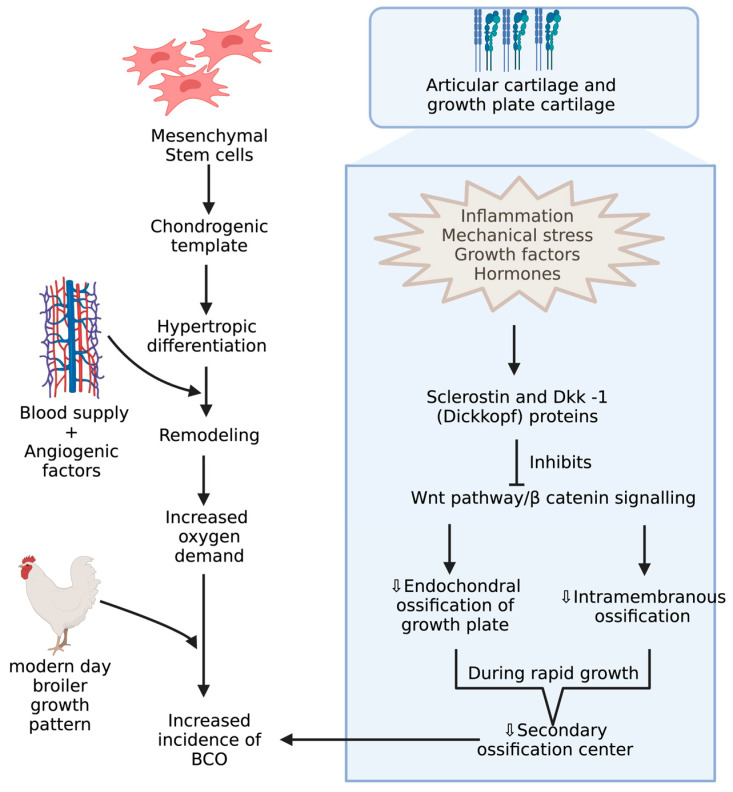
Mechanisms during pre and postnatal development of chicken increasing incidence of BCO.

BCO and other bone problems usually occur in the birds with higher growth rate and body gain which may be related to different breeds of commercial poultry [46,47]. Mostly, the body gain is attributed to pectoral muscles resulting in a shift in center of gravity with a disproportionate development of femur, which makes them more prone to BCO [48,49,50,51,52]. Integrity of articular cartilage (AC) and growth plate cartilages are crucial because the disproportionate development of legs to the body predisposes to injury under strenuous conditions. These cartilages greatly differ in their histology and extracellular matrices. AC is made of mostly all collagens except type X collagen and proteoglycans with chondrocytes [53,54]. Type X collagen appears in the growth plate usually when it undergoes the endochondral ossification process, which is assisted by adhesion molecules like cadherins and integrins, essential for regulating canonical signaling in Wnt pathway, which is activated upon binding of Wnt ligands to LRP-5/6 co receptors, and this can be inhibited when these receptors are bound to Wnt antagonists like sclerostin and Dkk-1 (Dickkopf proteins) [55,56,57](Figure 3). Among these adhesion molecules, cadherins mediate homotypic adhesion between bone cells, and integrins mediate adhesion between bone cells and its extracellular matrix [58]. Usually, long bones will develop by endochondral ossification where mesenchymal stem cells forms chondrogenic template through chondrogenic differentiation followed by hypertrophic differentiation, resulting in blood supply and remodeling of chondrogenic template into bone through the release of angiogenic factors [59]. Furthermore, the gradient increase in oxygen levels is also important in modulating the endochondral ossification where chondrogenic differentiation takes place at low levels of oxygen and hypertrophic differentiation at higher levels of oxygen tension [60,61]. The proximal tibial center is the only true secondary ossification center in the long bones of fowl during the rapid growth phase, leading to a reduced reinforcement of AC [62,63]. Although there is no conclusive evidence, ischemia is presumed to be the cause of lowered blood supply, leading to poor reinforcement of AC [19].

Osteochondrotic crypts are developed from poor mineralization of chondrocytes, resulting in microfractures that allow the opportunistic bacteria to colonize in those crypts through hematogenous routes [18,19,35,64,65,66,67]. These bacteria may have originated from broiler breeders, hatchery contamination, a gastrointestinal tract, a respiratory system, or an integumentary system [19,68,69]. In an experimentally induced and spontaneous occurring study on BCO in broilers, dyslipidemia was reported to be a common feature [70]. Moreover, thrombospondin, interferon γ, and transforming growth factor-ß, and angiogenesis inhibitors was suggested to be the risk of avascular necrosis [71,72]. In young chickens, the arrest of angiogenesis and growth plate development due to reduction in plasma levels of vascular endothelial growth factor isoform-C and protochaderin-15 (adhesion molecule) occurs in glucocorticoid-induced BCO, leading to apoptosis of chondrocytes [3,73]. Furthermore, reduced levels of fibroblast growth factor-2 and runt related transcription factor-2 (RUNX2), which are regulators of apoptosis and chondrocyte maturation, respectively, were suggestive of promoting BCO [74,75]. In some studies, serum metabolites like lipids, lipoproteins, and apolipoprotein-derived peptides showed changes when chickens were induced with glucocorticoids and naturally occurring BCO [3,70,76]. On the other hand, the use of these biomarkers for BCO needs further validation because they were significant in vascular diseases and osteoarthritis [77,78]. 

**Figure 4:**
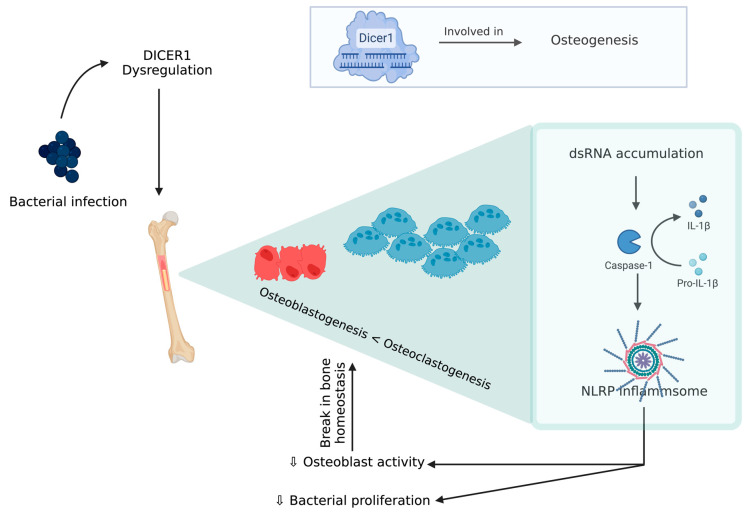
DICER 1 dysregulation in the presence of bacteria causing break in bone homeostasis.

An experimental model of chicken BCO for human osteomyelitis identified a novel pathophysiological mechanism for this severe inflammatory condition, which is described below [79]. DICER1 (a highly conserved RNaseIII endoribonuclease), a multifaceted protein which is responsible for dsRNA cleavage and its dysregulation, was recognized in several human diseases and was reported to have a critical role in osteogenesis [80,81,82,83,84,85]. DICER1 dysregulation alters cortical bone integrity and homeostasis which are usually associated with RUNX2 [86]. DICER1 dysregulation and infection exposure leads to an increase in dsRNA levels [79]. A study showed DICER1 dysregulation due to bacterial infection might induce dsRNA accumulation, which, in turn, is related to the IL-1β pathway, which plays a key role in pathogenesis of human bone inflammation. DICER1 dysmetabolism acts as an upstream regulator of NACHT (nucleotide-binding domain, LRR (leucine-rich repeat), and PYD (pyrin domain) domains containing protein (NLRP)3 inflammasome, upon activation of NLRP3, and paves the way for a break in bone homeostasis through the increased activity of neutrophils, monocytes, macrophages, osteoblasts, and osteoclasts [87,88,89,90,91] (Figure 4). Although there is a potential positive impact from inflammasome activation through a reduction in bacterial proliferation and removal of a pathogen from the host, this decreases the osteoblastic activity. Additionally, NLRP3 levels tend to be higher in bone tissue affected by a pathogen than the one with fractures, indicating that this inflammasome activation contributes to inflammatory bone loss [79,91]. MtDNA mutations are one form of mitochondrial dysfunction associated with alterations in mitochondrial biology [92,93]. This is usually associated with Alzheimer’s disease, dementia, coronary heart disease, chronic fatigue syndrome, and ataxia [92,93,94,95,96,97,98]. This association with several diseases is due to the relation between its dysfunction to apoptotic and inflammatory pathways. Mitochondria are direct targets for some bacterial infections like *Staphylococcus aureus* [96,99]. 

Peroxisome proliferator-activated receptor coactivator-1 (PGC-1α and PGC-1β) targets transcription factors (transcription factor A mitochondrial) and gene expression in mitochondrial biogenesis pathways [100]. Precisely, any changes in metabolism or cell growth will modulate the expression and upregulation of PGC-1α, leading to an increased mitochondrial biogenesis and respiration in inflammatory states [101,102]. Both mitochondrial biogenesis-associated genes (PGC-1α and PGC-1β) are significantly upregulated in BCO-affected tissue. In addition, the inflammatory response, associated reactive oxygen species accumulation, and metabolic shifts cause an increased need for mitochondria. On the other hand, during stress, mitochondrial fusion occurs, leading to formation of a large network, and this is associated with important components such as OPA1 (mitochondrial dynamin like GTPase) and Mitofusins (MFN1 and 2) [103,104]. Additionally, in ascites, OPA1 expression is decreased in the susceptible lines but not in ascites-resistant selected line [105]. In contrast, BCO showed a significant decrease in OPA1 but an upregulated MFN2 [106]. The decrease in the former is coupled with a gradual increase in OMA1 that is a regulator of mitochondrial fission via cutting OPA1 at some sites and making it inactive. At high levels of cellular stress, fission causes removal of damaged mitochondria when complementation through fusion is not possible. The above shift from fusion to fission indicates mitochondrial turn-over in accordance to the high level of stress during BCO [106]. Additionally, other potential and widely studied biomarkers are shown in Table 1.

## 5. Influences of Gut Microbiota in BCO

The commensal bacteria of the intestine that are acquired at perinatal stage are termed as gut microbiota [123]. The bacteria composition will stabilize by some period of time after birth; however, it varies from individual to individual through their diet, antibiotics, and infections [1]. This symbiotic relationship with the host helps in offering many antigens for the immune system. Dysbiosis in the gastrointestinal tract will lead to weakened immune system, making the host prone several diseases [1]. In addition, gut microbial niche plays a pivotal role in the pathogenesis of several diseases [124]. A study on mice grown in a germ-free environment showed a sterile gut and unfledged gut mucosal immune system; it also showed a reduction in T helper cells in spleen and peripheral blood, suggesting gut microbiota’s influence on systemic immunity development [124]. Furthermore, mice were protected from ovariectomy-induced bone loss. In these animals, bone mass and density are more with decreased bone resorption and regular bone formation [125]. This could be due to lesser number of T cells, proinflammatory, and pro-osteoclastogenic cytokines such as interleukin 6 (IL6) and tumor necrosis factor α (TNFα) [125]. Probiotic and prebiotic supplementation can enhance bone formation by upregulating SPARC (Osteonectin) and BMP-2 (Bome morphogenetic protein 2) genes involved in osteoblast formation [126]. In addition, probiotics ferment the prebiotics to short chain fatty acids (SCFA), reducing gut pH and abating the formation of calcium phosphates. Additionally, SCFAs influence calcium absorption through signaling pathway modulation and butyrate controls the calcium uptake by non-gut cells [127].

## 6. Miscellaneous Predisposing Factors for BCO

Calcium and phosphorus levels that are optimal for chicken feeding are 2:1 and are usually kept up by using fodder phosphates, fodder chalk, and enzymes like phytase. Imbalance in this ratio due to excessive supplementation of either of these macro elements leads to altered assimilation of other element. Water containing higher levels (>75 mg/L) of calcium affects the nutrient and medicine absorption [128]. The addition of 25-hydroxy vitamin D3 to water helps in alleviating calcium malabsorption [11]. In another study, supplementation of charcoal decreases the calcium bioavailability, leading to increased bone disorders due to increased phosphorus content in tibia [113]. In plants, there will be nearly 70% phosphorus in the form of phytic acid which can only be hydrolyzed by phytase [128]. Additionally, dietary supplementation of this enzyme enhances the tibial Mg and Fe concentration and also Zn utilization [129,130]. On the other hand, the action of this enzyme is influenced by gut microflora and enzymes wherein fibrinolytic enzymes has a synergistic effect with phytase. Furthermore, microelements like fluorine and boron are proven to be beneficial in attaining good bone density [128]. In contrast, suboptimal levels of copper in diet leads to shrinkage of collagen network and lowered bone mineral density. An increase in zinc to 100 mg/kg in feed leads to good bone strength and reduction in lameness in broiler chickens [131]. Additionally, vitamins like A, C, K apart from cholecalciferol affect maturation of chondrocytes, collagen synthesis, and ossification process, respectively. Additionally, supplementation of lysophospholipid improved intestinal development along with gut and bone health [132]. 

Moreover, feed quality, especially with reference to mycotoxins, possess a contradictory effect in terms of bird growth performance, health, and reproduction. Precisely, aflatoxin and ochratoxin have a negative influence on bone properties and allow the lameness problem to flourish from 2.3% to 25% [133]. Astonishingly, probiotic bacteria like *Bacillus licheniformis*, *B. subtilis*, and *Lactobacillus* spp. increased uptake of calcium, phosphorus, and bone inorganic substances, thus increasing the bone mechanical strength. Furthermore, dietary supplementation of probiotic, prebiotic, and synbiotics alleviate lameness concerns. 

Furthermore, bone development in poultry greatly rely on environmental conditions during incubation and production and on managerial factors like litter quality, lighting program, stocking density, ventilation, drinking water quality, and supplements. During incubation, an increase in temperature and lowered oxygen levels in the final phase of incubation leads to poor development of bones and type X collagen leading to asymmetric skeleton in broilers. Nowadays, embryos have a high metabolic rate wherein the bone growth rate is higher in last phase of incubation, which indicates that nutrient deficiency in this phase can cause an incompletely developed skeletal system and digestive system [50]. Numerous physiological changes occur during heat stress, leading to the production of glucocorticoids, which is reported to induce BCO [107,134].

**Figure 5:**
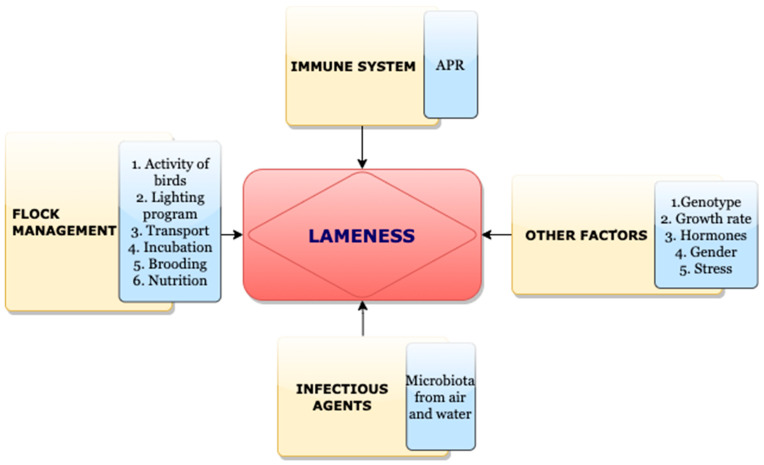
Factors affecting incidence of lameness.

A crucial factor in poultry production which correlated with bone pathologies is light intensity and ambient temperature. Intermittent lighting system is commonly reported to increase body mass and feed conversion. Additionally, there exists an interaction between the lighting program and sex of the bird. Male birds are more prone to leg health problems where a proper lighting schedule will be helpful. In addition, thermal stress is proved to reduce bone mass and its mechanical strength. Additionally, extremely low temperatures can decrease the mineral absorption. Furthermore, the presence of pathogenic bacteria is associated with temperatures. For instance, *E. coli* and *Enterococcus* have a highest prevalence in hot months compared to colder months. Additionally, poor gait scores tend to be more high in September but less so in March [128] (Figure 5).

## 7. Bacterial Diversities through Culture Independent Methods

Although some broilers would not exhibit clinical signs of lameness, there exists a progressive development of lesions that are pathognomonic to BCO. Sometimes, routine culture methods possess some cons, especially with respect to nonculturable species that remain undetected. Next generation sequencing which are culture-independent would help in identifying the bacterial communities by deep profiling of their 16s rRNA gene sequences. In a study, there was great diversity within a bacterial community in the initial days, but the trend decreased with aging and it was reversed in cecal samples [135]. *Staphylococcus aureus* was detected in culture-dependent and -independent methods. Furthermore, ultrastructural studies showed that this organism damages growth plate cartilage and proliferates within thick adherent glycocalyx. This does not allow the antibiotic penetration but presents the bacterial cell surface to host defense mechanisms [136]. Furthermore, Enterobacter, Serratia, and Nitrinicola were over presented in BCO samples, but these were not detectable through culture methods. Enterobacter and Serratia belong to family Enterobacteriaceae and Nitrinicola is an alkaliphilic bacterium. Serratia marcescens usually causes nosocomial infections and forms biofilms. Enterobacter strains are opportunistic pathogens. The same study indicated a great variability among individuals in the bacterial composition at various locations. Additionally, principal coordinate analysis showed the presence of individual specific selection pressures. In a study, bacterial communities that exist in the blood of chickens were analyzed. Although blood is considered to be sterile, it does contain immanent microbiota. In some studies, the most abundant phylum in blood of chicken was *Proteobacteria*. Other abundant phyla in the chicken blood were *Bacteroidetes*, *Firmicutes*, *Actinobacteria*, and *Cyanobacteria*. Furthermore, the most abundant phylum in chicken gut includes firmicutes, followed by *Proteobacteria* and *Bacteroidetes* [67]. These studies in different species infer that blood may lodge selected microbiota and possess a unique habitat to maintain them stably. In contrast, blood microbiota dysbiosis in any means may lead to significant increase of other phyla which might be due to leakage of gut microbiota under stress. An analysis from the above study showed the existence of 30 to 40 OTUs microbiota in the blood of broiler chickens, irrespective of the age and host factors [67]. Several analyses like beta diversity, hierarchical clustering, and bacterial network analysis suggested the existence of distinctive bacterial communities in BCO-affected birds from the healthy birds representing shift in these communities with certain selective pressures [67]. 

## 8. Mesenchymal Stem Cell Cultures—A Potential Tool to Understand BCO

Mesenchymal stem cells (MSCs) are spindle-shaped, adherent, non-hemopoietic stem cells lodges in bone marrow. They are usually isolated after removal of non-adherent cells from whole bone marrow aspirates followed by culturing adherent mononuclear layer in Dulbecco’s modified eagle medium (DMEM) supplemented with 10% fetal bovine serum. MSCs can be retrieved from the umbilical cord wall, blood, adipose tissue, liver, and skin [137]. In addition, characterization can be carried out using positive markers like CD44, Sca-1, CD71, CD73, CD90, and CD105. Negative markers include hematopoietic and endothelial markers like CD45, CD34, CD19, CD11b, CD11c, CD79a, and CD31, along with co-stimulatory molecules like CD80, CD86, and CD40 [137,138]. Tissue-specific phenotypes of MSCs are regulated by molecular signature linked to MSCs habitat. These are capable of differentiating into various cell types of mesodermal and non-mesodermal origin like chondrocytes, osteocytes, adipocytes, endothelial cells, cardiomyocytes, hepatocytes, and neural cells [139,140]. On the top, there are no standardized methods to isolate and identify differential populations, since they vary in basic and fundamental properties that are critical for differentiation. In an experimental design, 1,25-Dihydroxyvitamin D3 showed some inhibitory effects in initial stages of differentiation (1–2 days), but the latter stages were shown with stimulatory effects (3–7 days). This could be due to inhibition of RUNX2 and BMP2 expression in former stages [116,136].

A better way to understand BCO through culture models is by harboring MSCs. This can applied for several applications and aids in understanding mechanism of osteogenic, myogenic, and adipogenic pathways along with the compounds involved in driving them [137]. Furthermore, MSCs in culture conditions can adhere to a plastic surface and have multilineage differentiation property, and unveil the surface antigens [138]. Isolation of MSCs involves several purification steps since there are chances of contamination with blood cells and hematopoietic stem cells. In addition, the purification and enrichment of MSCs can be carried out by several methods like the use of ficole, antibody-based cell sorting, density-based culture techniques. In contrast, the isolation of MSCs from compact bones of day-old chickens could be an economical and easy process. Additionally, they demonstrated multilineage differentiation potential when provided with respective differentiation conditions. A study showed that MSCs and pericytes are developmentally related and share common phenotypic markers like CD146, NG2, and PDF-Rβ. CD146 expression attributes to osteogenic and immunomodulatory potential, along with hematopoietic control and therapeutic efficacy. In a rat model of acute inflammation of synovial membrane, intra-articular injection of POS cells promoted M2 macrophages polarization indicative of anti-inflammatory and healing mechanisms of synovium [138]. 20(S)-Hydroxycholesterol supplementation stimulates osteogenic differentiation accompanied with HES-1 and HEY-1 (Notch target genes) expression of MSCs through positive regulation of RUNX2. This occurs through the hedgehog signaling pathway, which is reported to be a key for bone homeostasis through coordinating maintenance of mesenchymal cell progenitors. LXR (Liver X receptor) signaling also plays a key role in HEY-1 expression in MSCs [140].

## 9. Conclusions

BCO turned out to be a major concern in poultry production and animal welfare. Understanding the underlying mechanisms involved in the incidence is very crucial in restraining this problem. Several studies can enhance our knowledge on BCO. However, some studies were not precise in tracing this knot. Through some experimental models, like the flooring models that can induce BCO lesions and cell culture models, divulging novel BCO mechanisms that further could help to arrive at a precise etiology along with diagnosis and treatment can take place. Although there were several studies related to BCO, there is a huge lacuna in finding precise biomarkers, pathophysiological mechanisms, and treatment alternatives. This would help in building a productive poultry industry which can resist challenges from genetic improvements like heavy body weights.

Finally, the genetic potential of modern-day broilers led them to gain more body weight but also made them more prone to skeletal issues. This damage is associated with opportunistic *pathogens* translocating to the skeletal tissues, affecting the bone turnover process, and commonly referred to as Bacterial Chondronecrosis and Osteomyelitis (BCO). Additionally, BCO is the leading cause of lameness in several countries, but information available on it is very limited. Furthermore, bone homeostasis disruption is associated with several pathways. BCO alters physiological processes, which are discussed in this manuscript, aids in targeting the potential steps in the pathways and helping to resolve the global economic and welfare concerns associated with the poultry industry. The focus on the anatomy, physiology, microbiota, and various pathogens associated with BCO, and potential ways to resolve the hidden pathways to untangle the locomotor problems in poultry. Furthermore, therapeutic, and prophylactic measures are currently available to combat this global problem for sustainable growth of broilers without compromising skeletal health.

## Figures and Tables

**Table 1:** Represents the potential biomarkers involved in the incidence of BCO.

Biomarkers	Function and Correlation	References
Serum Calcium	Bone density and mineralization along with bone breaking strength	[107,108]
IL-17, IL-6, TNF-α, NLRP-3	Pyroptosis of osteoblasts, and Pro-inflammatory factors stimulates osteoclastogenesis or inhibits osteobalstogenesis	[106,109]
Peroxisome proliferator actvated receptor coactivator (PGC-1α, 1β)	Repress the transcriptional activity of NF-κB, Mitochondrial biogenesis	[106,110,111]
Mitofusins	Increased ROS production,	[106]
Matrix metalloproteases	Tissue remodeling, angiogenesis, Extracellular matrix degradation	[107,112]
Osteocalcin	Secreted by differentiating osteoblasts	[14,75,113]
RANKL and OPG	Crtitical cytokine produced by osteoblasts and OPG is an decoy receptor for RANKL	[83,114,115]
Alkaline phosphatase	Involved in Ca and P deposition during the bone mineralization and formation	[9,111,116]
Sclerostin, DICKKOPF protein	Inhibit Wnt/β-catenin signalling pathway	[117,118,119,120]
Tartarate resistant Acid Phosphatase	Activity of osteoclasts	[19,109,111]
Thrombospondin, Interferon-γ, Tranforming growth factor-β, Vascular endothelial growth factor isoform-C, and Protocadherin-15	Associated with the risk of avascular necrosis seen in BCO	[9,19,69,121]
Fibroblast growth factor-2, BMP, SMAD1 and RUNX-2	Essential in osteoblast activity, bone mineralization, and osteoclast differentiation	[4,32,122]

## Data Availability

No new data were created in this study.
